# Heart and Skeletal Muscle Inflammation of Farmed Salmon Is Associated with Infection with a Novel Reovirus

**DOI:** 10.1371/journal.pone.0011487

**Published:** 2010-07-09

**Authors:** Gustavo Palacios, Marie Lovoll, Torstein Tengs, Mady Hornig, Stephen Hutchison, Jeffrey Hui, Ruth-Torill Kongtorp, Nazir Savji, Ana V. Bussetti, Alexander Solovyov, Anja B. Kristoffersen, Christopher Celone, Craig Street, Vladimir Trifonov, David L. Hirschberg, Raul Rabadan, Michael Egholm, Espen Rimstad, W. Ian Lipkin

**Affiliations:** 1 Center for Infection and Immunity, Columbia University, New York, New York, United States of America; 2 National Veterinary Institute, Oslo, Norway; 3 454 Life Sciences, Branford, Connecticut, United States of America; 4 Ministry of Fisheries and Coastal Affairs, Oslo, Norway; 5 Department of Biomedical Informatics and Center for Computational Biology and Bioinformatics, Columbia University, New York, New York, United States of America; 6 Norwegian School of Veterinary Science, Oslo, Norway; Yale University, United States of America

## Abstract

Atlantic salmon (*Salmo salar L.*) mariculture has been associated with epidemics of infectious diseases that threaten not only local production, but also wild fish coming into close proximity to marine pens and fish escaping from them. Heart and skeletal muscle inflammation (HSMI) is a frequently fatal disease of farmed Atlantic salmon. First recognized in one farm in Norway in 1999[1], HSMI was subsequently implicated in outbreaks in other farms in Norway and the United Kingdom[2]. Although pathology and disease transmission studies indicated an infectious basis, efforts to identify an agent were unsuccessful. Here we provide evidence that HSMI is associated with infection with piscine reovirus (PRV). PRV is a novel reovirus identified by unbiased high throughput DNA sequencing and a bioinformatics program focused on nucleotide frequency as well as sequence alignment and motif analyses. Formal implication of PRV in HSMI will require isolation in cell culture and fulfillment of Koch's postulates, or prevention or modification of disease through use of specific drugs or vaccines. Nonetheless, as our data indicate that a causal relationship is plausible, measures must be taken to control PRV not only because it threatens domestic salmon production but also due to the potential for transmission to wild salmon populations.

## Introduction

Fish are an increasingly important source of food and income; global annual consumption is projected to rise from 110 million tons in 2010 to more than 200 million tons in 2030. With depletion of wild stocks, suppliers are shifting from capture fishing to aquaculture. However, the emergence of infectious diseases in aquaculture threatens production and may also impact wild fish populations. Heart and skeletal muscle inflammation (HSMI), first detected in farmed Atlantic salmon (*Salmo salar L.*) in a single farm in Norway in 1999[1], has now been reported in 417 farms in Norway, as well as in the United Kingdom[2]. HSMI appears 5 to 9 months after fish are transferred from fresh water to ocean pens[1], is characterized by epi-, endo- and myocarditis, myocardial necrosis, myositis and necrosis of red skeletal muscle, and up to 20% mortality[3]. Disease can be induced in naïve fish by experimental injection with tissue homogenate from HSMI diseased fish or by cohabitation with fish with HSMI[1]. Virus-like particles have been observed[4]; however, efforts to implicate an infectious agent by using culture, subtractive cloning and consensus polymerase chain reaction for detection of other viruses found in salmon aquaculture including infectious pancreatic necrosis virus, salmonid alphavirus, infectious salmon anemia virus have been unsuccessful.

Here we provide evidence that HSMI is associated with infection with a novel reovirus. Piscine reovirus (PRV) was identified through high-throughput pyrosequencing of serum and heart tissue of experimentally infected fish using novel frequency analysis methods as well as standard alignment methods.

## Results

RNA extracted from heart of a salmon with experimentally induced HMSI was pyrosequenced[5] yielding 106,073 reads ranging in size up to 598 nucleotide (average = 349.7, SD = 149.5). Although database alignment analysis at the nucleotide level revealed no evidence of infection, the predicted amino acid sequence of one 265 nucleotide read was 49% similar to the core-spike protein λ2 of Mammalian orthoreovirus 3 (AF378009). A real time PCR assay based on this sequence was used to test for the presence of the candidate virus in RNA extracts of heart and serum obtained from salmon with HSMI in association with spontaneous outbreaks (n = 20) or experimental infection (n = 20), and in non-infected control fish (n = 20). All samples from salmon with HSMI contained the candidate sequences. No sequences were found in the control salmon without HSMI.

**Figure 1:**
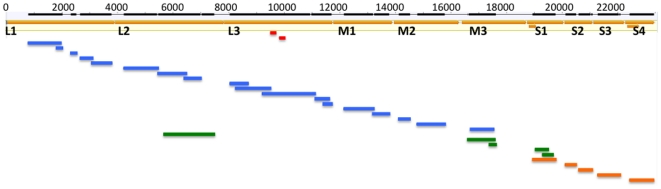
Piscine reovirus (PRV) sequence obtained by pyrosequencing. Assembled sequence data mapped against the concatenated sequences of PRV. Genomic regions identified by BLASTN, BLASTX, FASTX, and FASD are shown in red, blue, green, and orange respectively.

The HSMI serum sample with the highest genetic load by PCR (3.0×10^6^ genome copies/µl) was selected for additional pyrosequencing yielding 120,705 reads. A suite of bioinformatic tools was used to identify viral sequences. In the first phase of analysis, BLASTN and BLASTX[6] detected 1.5% and 53.9% of the predicted viral genome, respectively, enabling identification of segments L1, L2, L3, M1, M2 and M3 (**Figure 1**). Implementation of FASTX[7] yielded an additional 5.5% of the genome and detected motifs in the S1 segment as well as additional sequences in the L2 and M3 segments. Frequency Analysis of Sequence Data (FASD[8]), a program that predicts taxonomy based on nucleotide frequency and order rather than sequence alignment, detected new sequences representing the S1, S2, S3 and S4 segments (**Figure 1**) that comprised an additional 11.8% of the final viral genome assembly. In total, approximately 17 kilobases of sequence (72.8% of the genome) was obtained by pyrosequencing (**Figure 1**). Gaps between fragments and the termini of gene segments were completed by PCR cloning. All sequence was verified by classical dideoxy sequencing by using primers designed along the draft sequence.

**Figure 2:**
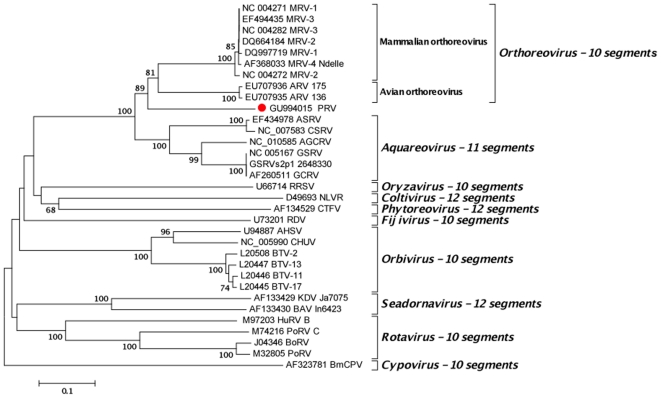
Phylogenetic analysis of the RNA-dependent RNA-polymerase of *Reoviridae*. Full length amino acid sequences were aligned using the ClustalX[18] implemented on MEGA software[19] and refined using T-Coffee[20] to incorporate protein structure data. Phylogenetic analysis was performed using p-distance as model of amino acid substitution as implemented by ICTV for analysis of the Reoviridae family[22]. MEGA was used to produce phylogenetic trees, reconstructed through the Neighbor Joining (NJ) method. The statistical significance of a particular tree topology was evaluated by bootstrap re-sampling of the sequences 1000 times.

Consistent with the genome organization characteristic for members of the family *Reoviridae*, the genome of the PRV comprises at least 10 RNA segments (GenBank Accession numbers GU994013-GU994022). Reoviruses are non-enveloped icosahedral viruses with double-stranded RNA genomes comprising 10–12 segments. Twelve genera are defined based on host range, number of genome segments, G/C content, and antigenic relationships. A phylogenetic tree constructed using L gene segment sequences of known reoviruses indicate that PRV represents a distinct genetic lineage branching off the root of the aquareovirus and orthoreovirus genera, viruses of fish and shellfish, reptiles, birds and mammals (**Figure 2**). Analysis of all ten PRV gene segments confirmed the divergence of PRV sequence with respect to other reoviruses (**Figures S1** **,** **S2** **,** **S3** **,** **S4** **,** **S5** **,** **S6** **,** **S7** **, and** **S8**). All PRV gene segments contained the 3′ terminal nucleotides (UCAUC-3′) found in orthoreoviruses and aquareoviruses[9]; however, the 5′ terminal nucleotides (5′-GAUAAA/U) were unique.

The orthoreoviruses have polycistronic segments in either S1 or S4. Whereas aquareovirus species C are polycistronic in the S7 (the orthoreovirus S1 homolog), the other aquareovirus species are not[9]. PRV has a putative open reading frame (ORF) in the 5′-end of S2 (71 aa, pI = 8.8, 8 kDA), and a putative ORF in 5′-end of S1 (124 aa, pI = 4.8, 13 kDA). Although homologues of the λ1, λ2, λ3, µ1, µ2, µ3, σ2 and σNS sequences of PRV are found in orthoreoviruses and aquareoviruses, the σ1 and σ3 sequences and the small putative open reading frames observed in S2 and S1 appear distinctive. The hydrophobicity plot of the latter is similar to a fusion-associated small transmembrane (FAST) reovirus protein[10] (**Figure S9**). Reovirus FAST proteins are nonstructural, single-pass membrane proteins that induce cell-cell fusion and syncytium formation[10]. Taken together these data provide compelling evidence that PRV is the prototype of a new reovirus genus equally distant to the orthoreovirus and aquareovirus genera.

**Figure 3:**
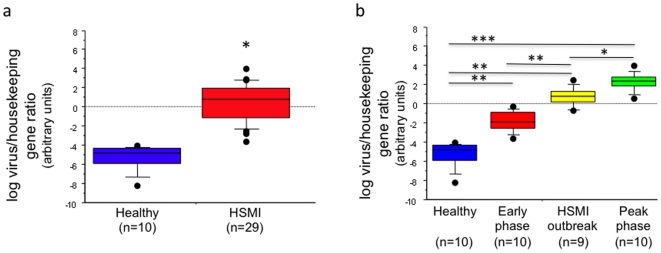
Graphical representation of group differences in the log ratio of virus load normalized to a salmon host gene. Nonparametric approaches were used to determine statistical significance for comparisons of the relative viral load among healthy and HSMI-affected farmed fish. Log transformations, which did not normalize log ratio distributions, were nonetheless performed for all samples after calculating L1 (virus)/EF1A (housekeeping) ratios to aid in graphical representation. **(a)** Comparison of adjusted log ratio in mixed heart and kidney samples from healthy farmed fish and farmed fish with HSMI; *, *p*<0.0001 (Mann-Whitney U); **(b)** comparison of adjusted log ratios in farmed fish without HSMI (healthy farmed fish), in the early phase of an HSMI outbreak, in the middle of an HSMI outbreak, and during the peak of an HSMI outbreak; **, *p*<0.0005; *, *p<*0.01 (individual Mann-Whitney U). Adjusted log ratios also differed significantly across all four farmed fish groups (*p*<0.0001; Kruskal-Wallis).

PRV burden in farmed and wild salmon was examined using real time PCR assays targeting genome segments L1, L2, M3 and S4. Levels of viral RNA were quantitated using an MGB assay against L1 wherein results were normalized to the host mRNA encoding elongation factor 1A (EF1A), using the formula by Pfaffl[11]. We studied heart and kidney samples from 29 salmon representing three different HSMI outbreaks **(Table S1)** and 10 samples from healthy farmed fish. Twenty-eight of the 29 (96.5%) known HSMI samples and none of the 10 (0%) healthy salmon samples were positive as defined by L1/EF1A gene log ratio≥5.00. Only one of 29 HSMI samples was negative; this sample originated from a salmon net harboring fish in the early phase of HSMI, prior to the onset of fish mortality (**Figure 3**). In fish with signs of severe disease, including abnormal swimming behavior, anorexia and histologic evidence of pancarditis and myositis[12], the median adjusted L1/EF1A gene log ratio was 10.36 (IQR, 0.94). The L1/EF1A gene log ratio was correlated not only with the presence or absence of HSMI, but also, with severity of disease at the time of sampling. The log ratios were lowest in healthy farmed salmon (log ratio range, −0.23 to 3.89; n = 10), higher in salmon collected in the early phase of an HSMI outbreak (range, 4.34 to 7.66; n = 10), and highest in salmon obtained at the peak of an HSMI outbreak (range, 8.52 to 11.90; n = 10). To study the prevalence and relative levels of PRV in healthy wild salmon from different geographic locations, we tested 66 samples obtained from nine coastal rivers in Norway. PRV was detected in only sixteen of these samples (24.2%). Two of these sixteen samples were positive by the cutoff established for farmed salmon with relative log ratios of 6.70 and 7.58; the other fourteen had L1/EF1A log ratios well below the 5.00 cutoff (range, −.20 to 4.57). No PRV transcripts were detected in any of the remaining wild salmon samples (n = 50).

**Figure 4:**
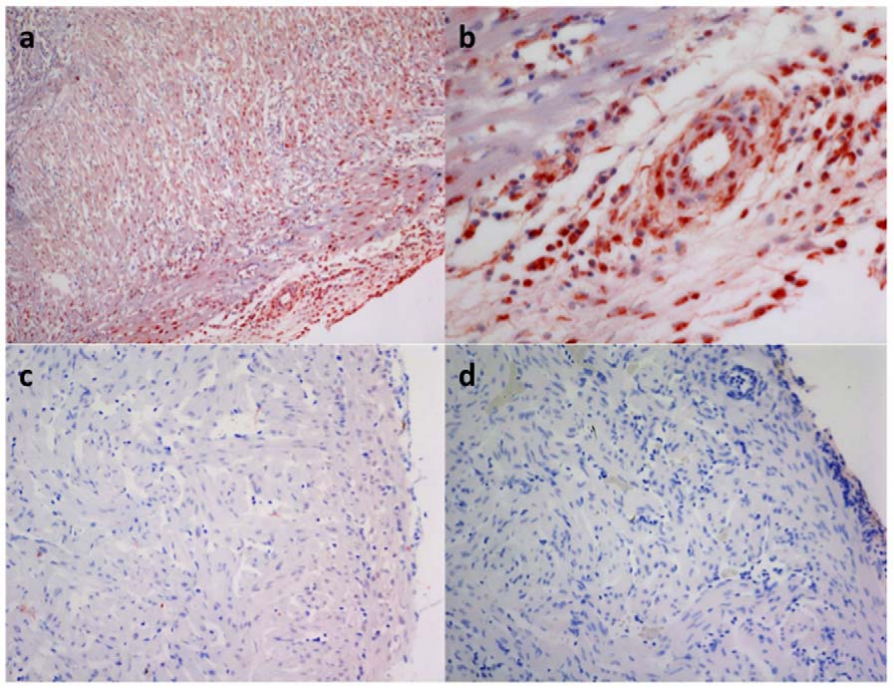
*In situ* hybridization was performed using locked nucleic acid (LNA) probes targeting the L2 segment of the Piscine reovirus. Sections were permeabilized using proteinase K followed by hybridisation with digoxigenin (DIG)-labeled LNA probes. Sections were incubated with a mouse monoclonal anti-DIG-horse radish peroxidase and stained using a Tyramide Signal Amplification System. Sections were counterstained with Meyer's hematoxylin solution. (a) Heart from HSMI-infected fish (10×); (b) heart from HSMI-infected fish (40×); (c) heart from non-infected fish (40×); (d) heart from a fish infected with salmon pancreas disease virus.

The anatomic distribution of PRV in relation to pathology was tested through *in situ* hybridization using probes to L2 gene RNA. PRV RNA was distributed throughout the myocardium and endocardium of salmon with HSMI (**Figure 4a, b**) but not detected in normal salmon or salmon infected with salmon pancreas disease virus (**Figure 4c, d**).

## Discussion

Implication of a microbe in a disease via Koch's postulate requires demonstration that an agent is specific for that disease, and that disease can be reproduced in a naïve host by inoculation with the agent propagated in culture following isolation from an affected host. Although fulfillment of this postulate is compelling evidence of causation the criteria are unduly stringent. Some agents cannot be cultured. Some infectious diseases, particularly those of humans, cannot be modeled due to ethical considerations. Additionally, genetic and other factors may contribute to pathogenesis. PRV has not been cultured. Furthermore, PRV has been found in farmed fish that do not show clinical signs of HSMI. Moreover, PRV has been also detected in low quantities in wild Atlantic salmon. Nonetheless, the tissue distribution and load of PRV are correlated with disease in naturally and experimentally infected salmon. Analogies between commercial poultry production and Atlantic salmon aquaculture may be informative. Reoviruses are also implicated in numerous diseases of poultry, including enteritis, myocarditis, and hepatitis[13]. Both poultry production and aquaculture confine animals at high density in conditions that are conducive to transmission of infectious agents and may reduce resistance to disease by induction of stress.

Unlike terrestrial animal farming, where contact between domestic and free ranging wild animals of the same or closely related species is easily monitored and controlled, ocean based aquaculture is an open system wherein farmed fish may incubate and transmit infectious agents to already diminishing stocks of wild fish. Formal implication of PRV in HSMI will require isolation in cell culture and fulfillment of Koch's postulates, or prevention or modification of disease through use of specific drugs or vaccines. Nonetheless, as our data indicate that a causal relationship is plausible, it is urgent that measures be taken to control PRV not only because it threatens domestic salmon production but also due to the potential for transmission to wild salmon populations.

## Materials and Methods

HSMI was experimentally-induced in normal Atlantic salmon by inoculation with heart and kidney extracts from fish with HSMI or cohabitation with fish with HSMI. Tissue specimens were homogenized, diluted 1∶10 in Leibowitz' L-15 cell culture medium (Sigma-Aldrich, St Louis, MO), and centrifuged at 2500 g for 10 min. The supernatant was diluted 1∶2 in L-15 containing gentamycin (final concentration 50 µg mL-1) prior to inoculation.”

RNA extracted from heart tissue from Atlantic salmon with experimentally-induced HSMI was used as template for high throughput pyrosequencing. Sequences were analyzed using a suite of bioinformatic applications available at the GreenePortal website (http://tako.cpmc.columbia.edu/Tools), including FASD, a method whereby the statistical distribution of oligonucleotide frequencies within an unknown sequence set is compared to frequencies calculated for known sequence sets. Seven of ten segments of a novel reovirus, piscine reovirus (PRV), were identified using alignment and a motif-based program; three additional segments were identified using FASD. Quantitative real time PCR assays of samples from fish collected during outbreaks of HSMI and from fish with experimentally-induced HSMI confirmed association between PRV and HSMI. *In situ* hybridization confirmed the presence of PRV sequences in heart of fish with HSMI.

### Identification of PVR by high-throughput sequencing

Healthy Atlantic salmon produced at an experimental facility (VESO, Vikan; Namsos, Norway), with an average weight of 50 g were inoculated with cardiac tissue from field outbreaks of HSMI and served as donors for material for the high-throughput sequencing. Non-inoculated fish served as negative controls[14].

Three heart muscle biopsies were diluted 1∶10 in HBSS, filtrated through a 0.22 µm filter and inactivated in TRIzol LS reagent (Invitrogen, Carlsbad, CA, USA). Several serum samples were inactivated directly in TRIzol LS. Total RNA extracts were treated with DNase I (DNA-free, Ambion, Austin, TX, USA) and cDNA generated by using the Superscript II system (Invitrogen) for reverse transcription primed by random octamers that were linked to an arbitrary defined 17-mer primer sequence[15]. The resulting cDNA was treated with RNase H and then randomly amplified by the polymerase chain reaction (PCR); applying a 9∶1 mixture of a primer corresponding to the defined 17-mer sequence and the random octamer-linked 17-mer primer, respectively[15]. Products >70 base pairs (bp) were selected by column purification (MinElute, Qiagen, Hilden, Germany) and ligated to specific linkers for sequencing on the 454 Genome Sequencer FLX (454 Life Sciences, Branford, CT, USA) without fragmentation of the cDNA[5], [16], [17]. Removal of primer sequences, redundancy filtering, and sequence assembly were performed with software programs accessible through the analysis applications at the GreenePortal website (http://156.145.83.115/Tools). When traditional BLASTN, BLASTX and FASTX analysis failed to identify the origin of the sequence read, we applied FASD[8], a novel method based on the statistical distribution of oligonucleotide frequencies. The probability of a given segment to belong to a class of viruses is computed from their distribution of oligonucleotide frequencies in comparison with the calculated for other segments. A statistic measure was developed to assess the significance of the relation between segments. The p-value estimates the likelihood that an oligonucleotide distribution is derived from a different one. Thus, highly related distributions present a high p-value.

Conventional PCRs were performed with HotStar polymerase (Qiagen) on PTC-200 thermocyclers (Bio-Rad, Hercules, CA, USA): an enzyme activation step of 5 min at 95°C was followed by 45 cycles of denaturation at 95°C for 1 min, annealing at 55°C for 1 min, and extension at 72°C for 1 to 3 min depending on the expected amplicon size. Amplification products were run on 1% agarose gels, purified (MinElute, Qiagen), and directly sequenced in both directions with ABI PRISM Big Dye Terminator 1.1 Cycle Sequencing kits on ABI PRISM 3700 DNA Analyzers (Perkin-Elmer Applied Biosystems, Foster City, CA).

### Sequence Analyses

Programs of the Geneious package (Biomatters, New Zealand) were used for sequence assembly and analysis. Sequences were downloaded from GenBank and aligned using the ClustalX[18] implementation on the MEGA software[19]. The amino acid alignments obtained were further refined using T-Coffee[20] to incorporate protein structure data on the alignment. To evaluate the robustness of the approach, the ability to find and align motifs previously identified as conserved among *Reoviridae* was used as a marker. Phylogenetic analysis were performed using p-distance as model of aminoacid substitution as accepted by ICTV for analysis of the *Reoviridae* family. MEGA was used to produce phylogenetic trees, reconstructed through the Neighbor Joining (NJ) method. The statistical significance of a particular tree topology was evaluated by bootstrap re-sampling of the sequences 1000 times. Bayesian phylogenetic analyses of the sequence differences among segments λ1, λ2, λ3, µ1, µ2, µ3, σ2 and σ3 (σ1 and σNS of aquareovirus and orthoreovirus had different genomic organizations) were conducted using the BEAST, BEAUti and Tracer analysis software packages. Preliminary analyses were run for 10,000,000 generations with the Dayhoff aminoacid substitution model to select the clock and demographic models most appropriate for each ORF. An analysis of the marginal likelihoods indicated that the relaxed lognormal molecular clock and constant population size model was chosen for all datasets. Final data analyses included MCMC chain lengths of 5,000,000–30,000,000 generations, with sampling every 1000 states (**Figures S1** **,** **S2** **,** **S3** **,** **S4** **,** **S5** **,** **S6** **,** **S7** **, and** **S8**).

### Real Time PCR

Quantitative assays were established based upon virus specific sequences obtained from the high throughput sequencing for several reovirus segments. Six different real-time assays were designed targeting genome fragment L1, L2 and M3 (SYBR green) as well as L1 and S4 (MGB assays) (primers available on request). Samples from different organs from experimentally infected fish were positive while samples from non-infected control fish were negative. For further screening, the real-time PCR for segment L1 was performed using the QIAGEN OneStep kit. Sixµl of template RNA were denatured (95°C/5 min). Reactions were performed using the following concentrations: 400 nM primer, 300 nM probe and 1.25 mM MgCl_2_. Amplifications were done in a Stratagene Mx3005P real-time PCR machine (Stratagene) with the following cycle parameters: 30 min at 50°C (reverse transcription), 15 min at 94°C (RT inactivation and PCR polymerase activation), 45 cycles of 94°C/15 sec, 54°C/30 sec and 72°C/15 sec. Standard curves were created using RNA pooled from three fish with high viral loads. Standard curves were made in duplicates for both the MGB assay and the EF1A assay[21] and relative viral RNA loads for field samples were calculated by using normalization against EF1A.

### *In situ* hybridization

*In situ* hybridization was performed in compliance with the protocol from GeneDetect (Auckland, New Zealand) with some modifications using LNA probes (Exiqon, Vedbaek, Denmark) targeting L2. Sections were permeabilized using 40 µg ml^−1^ Proteinase K (Novagen, WI, USA) in TE buffer at 37°C for 15 min followed by hybridization with a mixture of two 5′ and 3′ double DIG labelled LNA probes (5′-CACCATCAGTGAACTTAGGAGCAAC-3′ and 5′-CATACTCCAAGATCATCGCCAGCA-3′) (250 nM each) for 18 hours at 50°C. Stringency washes were carried out at 60°C. Sections were incubated with a mouse monoclonal anti-DIG-HRP (Abcam, Cambridge, UK) overnight at 4°C and stained using a Tyramide Signal Amplification System (Perkin Elmer, MA, USA) according to the manufacturer's protocol. Sections were counterstained with Meyer's hematoxylin solution (AppliChem, Darmstadt, Germany). Negative controls included were samples from non-infected fish from experimental trial, head kidney samples from non-infected fish as a source of immune cells, salmon with pancreatic disease (a differential diagnosis to HSMI), and samples from material sent for diagnostics at random.

### Statistical analysis

StatView version 5.0.1 software for Windows (SAS Institute, Cary, NC, USA) was used for all statistical analyses. Samples without detectable L1 viral gene transcripts were excluded from statistical analysis. Log transformations were performed for all other samples after calculating L1/EF1A ratios (adjusted by a factor of 10^8^). Log-transformed data were retained to facilitate graphical display of group differences, though distributions were not normalized by this method; thus, nonparametric analytic approaches were employed (Mann–Whitney U-test for comparison of healthy and HSMI fish; Kruskal-Wallis for comparisons of healthy and early, middle and peak phase HSMI fish). For all tests, statistical significance was assumed where *p*<0.05.

## Supporting Information

Figure S1 Phylogenetic analysis of the Lambda-1 ORF of the Aquareovirus and Orthoreovirus. Phylogenetic analysis of the Lambda-1 ORF of the Aquareovirus and Orthoreovirus. Bayesian phylogenetic analyses of sequence differences among segments λ1, λ2, λ3, µ1, µ2, µ3, σ2 and σNS (σ1 and σ3 of aquareovirus and orthoreovirus had different genomic organizations) were conducted using BEAST, BEAUti and Tracer analysis software packages. Preliminary analyses were run for 10,000,000 generations with the Dayhoff aminoacid substitution model to select the clock and demographic models most appropriate for each ORF. An analysis of the marginal likelihoods indicated that the relaxed lognormal molecular clock and constant population size model was chosen for all datasets. Final data analyses included MCMC chain lengths of 5,000,000 - 30,000,000 generations, with sampling every 1000 states (Figure S1–S8). Colored boxes indicate representatives of different reovirus genera or species. Green, Aquareovirus genus; blue, species I (mammalian orthoreovirus); red, species II (avian orthoreovirus); purple, species III (Nelson Bay orthoreovirus); orange, species IV (reptilian orthoreovirus) and light blue, species V (Baboon orthoreovirus).
(3.00 MB TIF)

Figure S2 Phylogenetic analysis of the Lambda-2 ORF of the Aquareovirus and Orthoreovirus. For methods and notations, see Figure S1 legend.
(3.00 MB TIF)

Figure S3 Phylogenetic analysis of the Lambda-3 ORF of the Aquareovirus and Orthoreovirus. For methods and notations, see Figure S1 legend.
(3.00 MB TIF)

Figure S4 Phylogenetic analysis of the Mu-1 ORF of the Aquareovirus and Orthoreovirus. For methods and notations, see Figure S1 legend.
(3.00 MB TIF)

Figure S5 Phylogenetic analysis of the Mu-2 ORF of the Aquareovirus and Orthoreovirus. For methods and notations, see Figure S1 legend.
(3.00 MB TIF)

Figure S6 Phylogenetic analysis of the Mu-3 ORF of the Aquareovirus and Orthoreovirus. For methods and notations, see Figure S1 legend.
(3.00 MB TIF)

Figure S7 Phylogenetic analysis of the Sigma-2 ORF of the Aquareovirus and Orthoreovirus. For methods and notations, see Figure S1 legend.
(3.00 MB TIF)

Figure S8 Phylogenetic analysis of the Sigma-NS ORF of the Aquareovirus and Orthoreovirus. For methods and notations, see Figure S1 legend.
(3.00 MB TIF)

Figure S9 Putative ORF of S1 has characteristics similar to FAST proteins. Hydrophobicity plots of ARV (red) and PRV (blue) obtained using the Kyle-Doolittle algorithm implemented in the program TopPred (available at http://mobyle.pasteur.fr/cgi-bin/portal.py?form=toppred). Sequence analysis show that PRV contains the primary components of a FAST protein: hydrophobic region (HP), transmembrane domain (TM) and basic region (BR).
(3.00 MB TIF)

Table S1 a Ratio of virus burden (quantitated through the L1 viral gene), normalized using a salmon housekeeping gene (EF1A) and adjusted by a factor of 108 b Log transformation of the adjusted ratio L1/EF1A c Virus detection by real time RT-PCR d For statistical analyses, samples were considered positive whenever the adjusted log ratio was higher than 5.00
(0.03 MB XLS)
