# Supplementary figures and images for: Heart and Skeletal Muscle Inflammation of Farmed Salmon Is Associated with Infection with a Novel Reovirus
Source: PLoS One. 2010 Jul 9;5(7):e11487. doi: 10.1371/journal.pone.0011487 (PMC2901333; doi:10.1371/journal.pone.0011487)

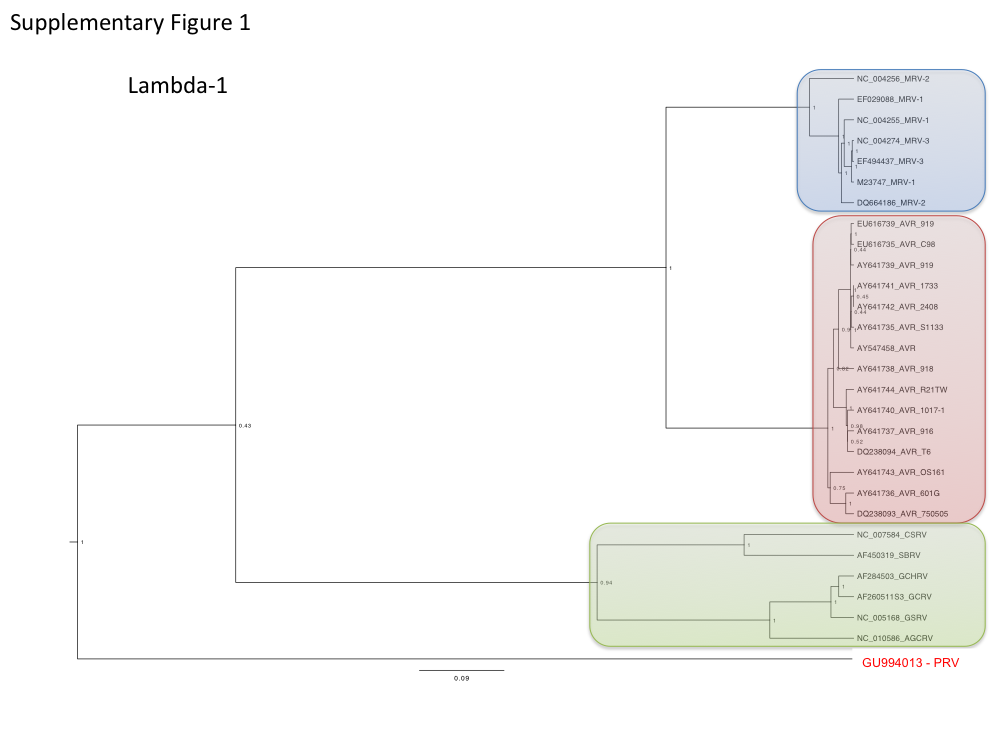

Supplement: Figure S1 — Phylogenetic analysis of the Lambda-1 ORF of the Aquareovirus and Orthoreovirus. Phylogenetic analysis of the Lambda-1 ORF of the Aquareovirus and Orthoreovirus. Bayesian phylogenetic analyses of sequence differences among segments λ1, λ2, λ3, µ1, µ2, µ3, σ2 and σNS (σ1 and σ3 of aquareovirus and orthoreovirus had different genomic organizations) were conducted using BEAST, BEAUti and Tracer analysis software packages. Preliminary analyses were run for 10,000,000 generations with the Dayhoff aminoacid substitution model to select the clock and demographic models most appropriate for each ORF. An analysis of the marginal likelihoods indicated that the relaxed lognormal molecular clock and constant population size model was chosen for all datasets. Final data analyses included MCMC chain lengths of 5,000,000 - 30,000,000 generations, with sampling every 1000 states (Figure S1–S8). Colored boxes indicate representatives of different reovirus genera or species. Green, Aquareovirus genus; blue, species I (mammalian orthoreovirus); red, species II (avian orthoreovirus); purple, species III (Nelson Bay orthoreovirus); orange, species IV (reptilian orthoreovirus) and light blue, species V (Baboon orthoreovirus). (3.00 MB TIF) [file pone.0011487.s001.tif]

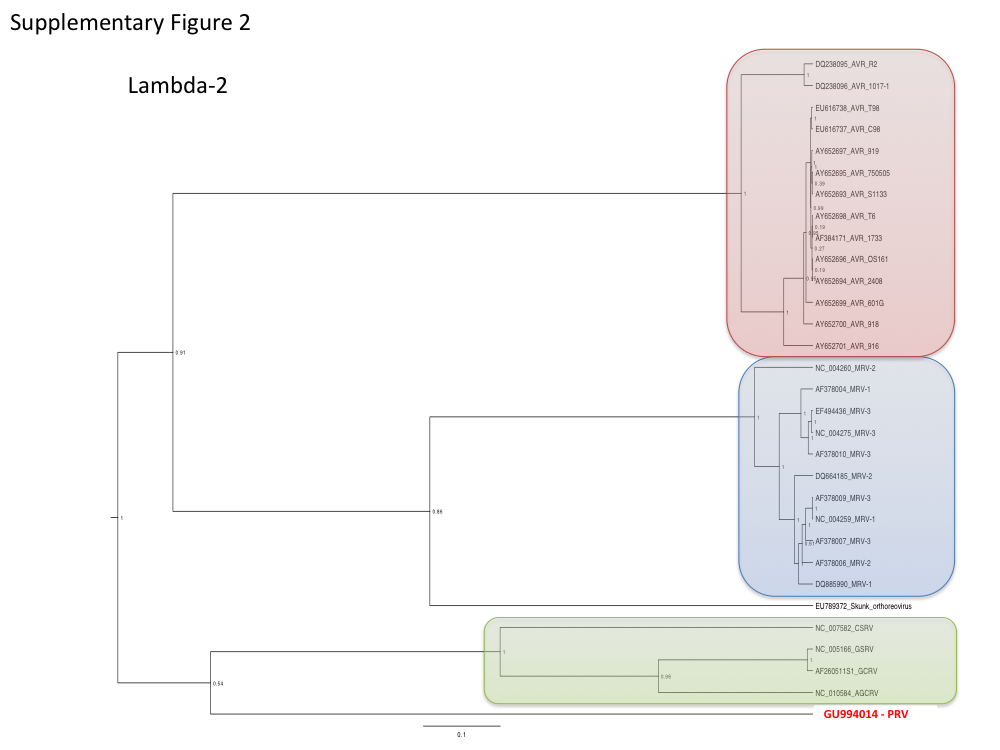

Supplement: Figure S2 — Phylogenetic analysis of the Lambda-2 ORF of the Aquareovirus and Orthoreovirus. For methods and notations, see Figure S1 legend. (3.00 MB TIF) [file pone.0011487.s002.tif]

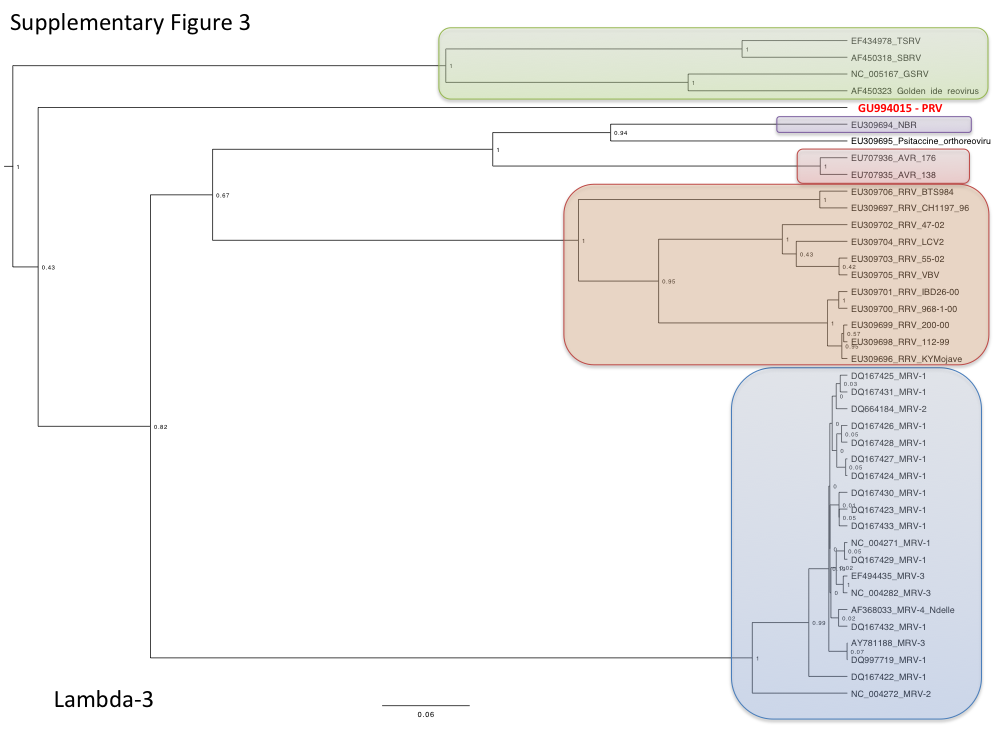

Supplement: Figure S3 — Phylogenetic analysis of the Lambda-3 ORF of the Aquareovirus and Orthoreovirus. For methods and notations, see Figure S1 legend. (3.00 MB TIF) [file pone.0011487.s003.tif]

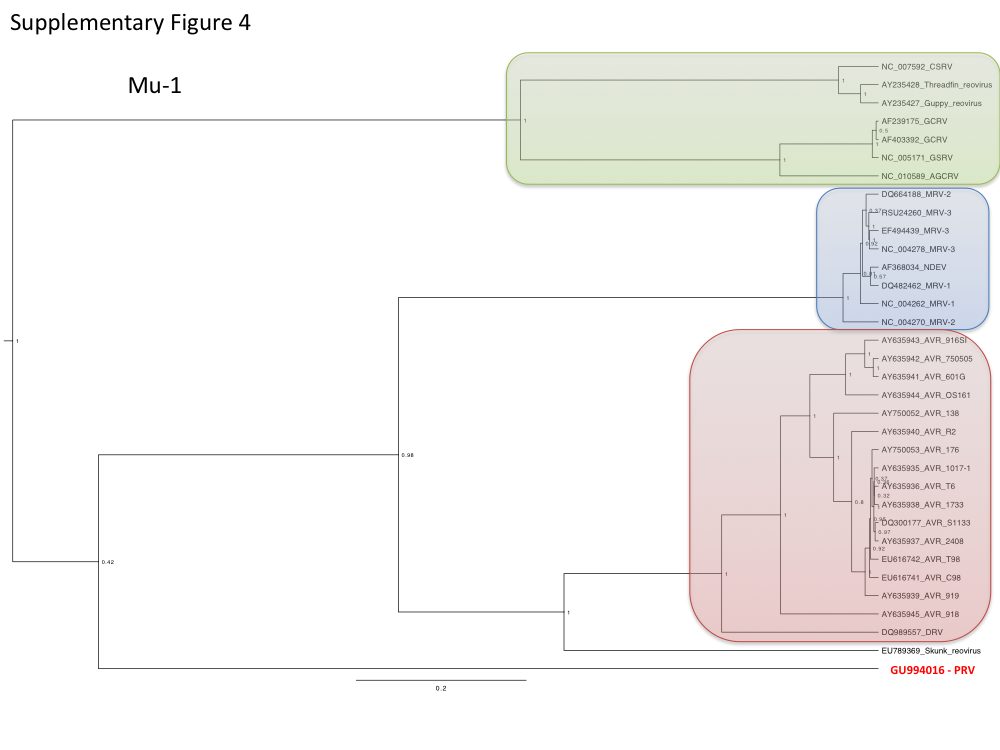

Supplement: Figure S4 — Phylogenetic analysis of the Mu-1 ORF of the Aquareovirus and Orthoreovirus. For methods and notations, see Figure S1 legend. (3.00 MB TIF) [file pone.0011487.s004.tif]

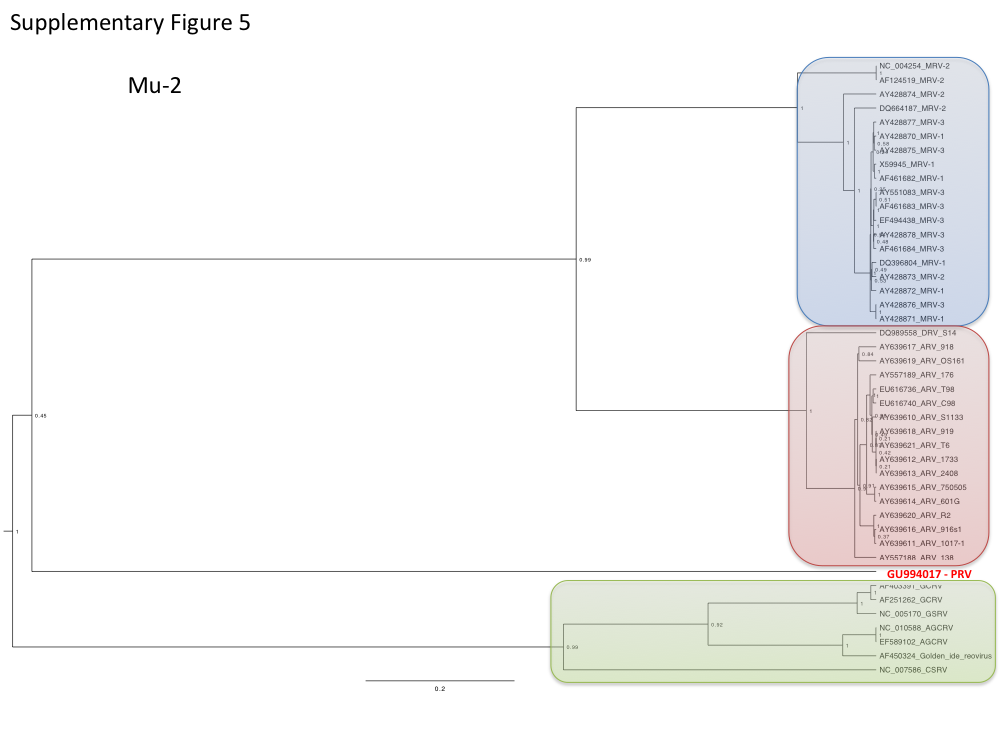

Supplement: Figure S5 — Phylogenetic analysis of the Mu-2 ORF of the Aquareovirus and Orthoreovirus. For methods and notations, see Figure S1 legend. (3.00 MB TIF) [file pone.0011487.s005.tif]

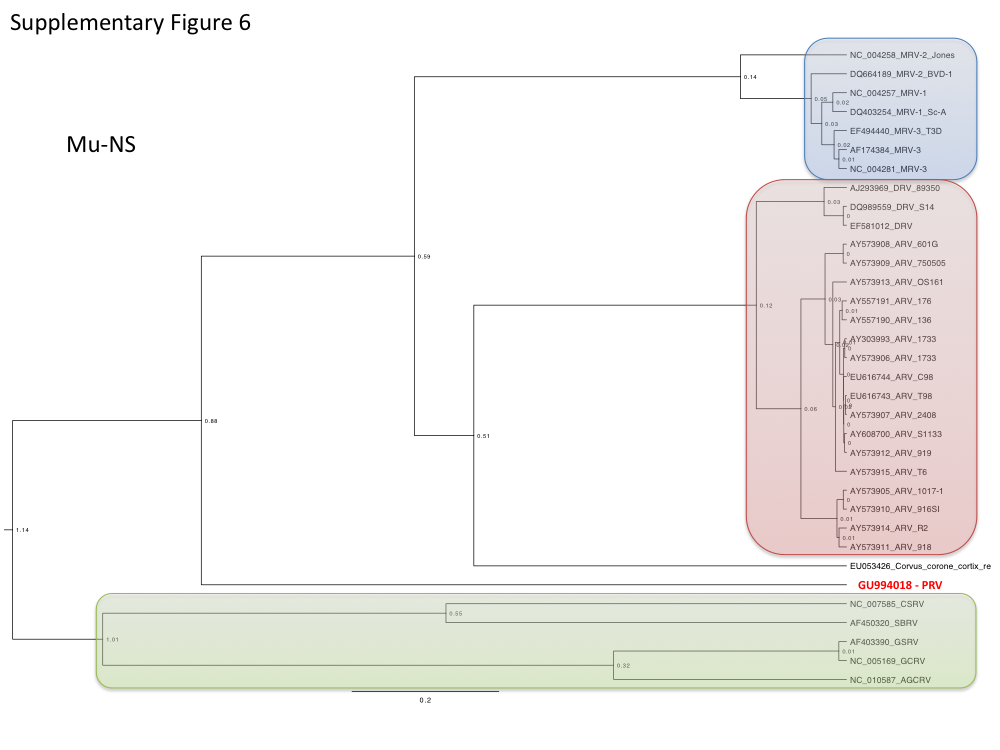

Supplement: Figure S6 — Phylogenetic analysis of the Mu-3 ORF of the Aquareovirus and Orthoreovirus. For methods and notations, see Figure S1 legend. (3.00 MB TIF) [file pone.0011487.s006.tif]

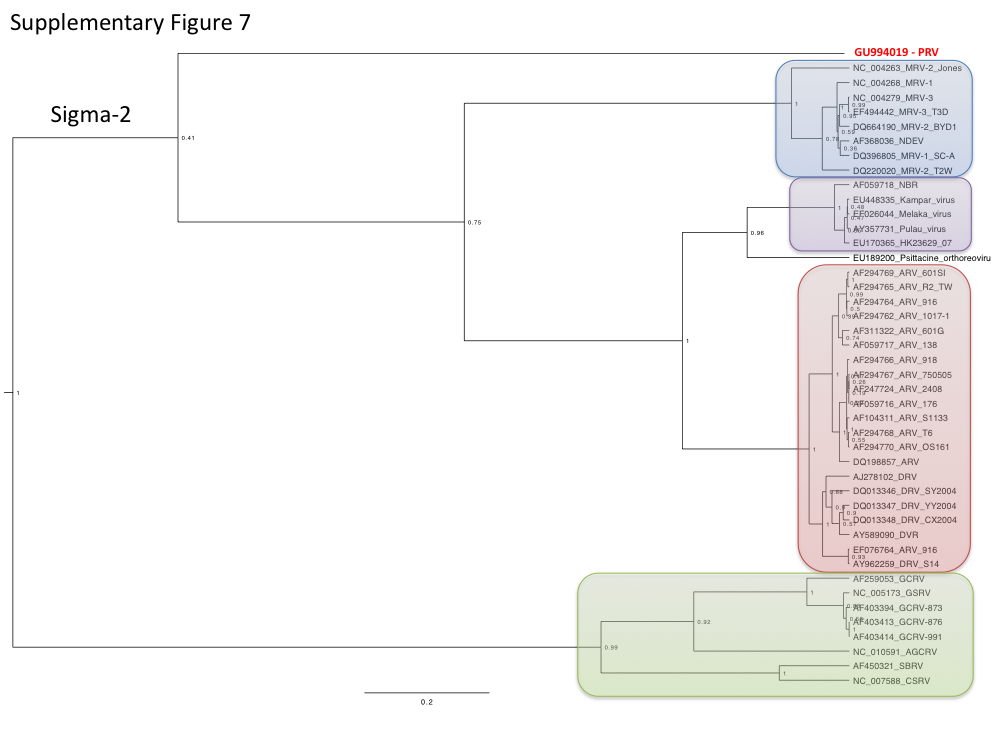

Supplement: Figure S7 — Phylogenetic analysis of the Sigma-2 ORF of the Aquareovirus and Orthoreovirus. For methods and notations, see Figure S1 legend. (3.00 MB TIF) [file pone.0011487.s007.tif]

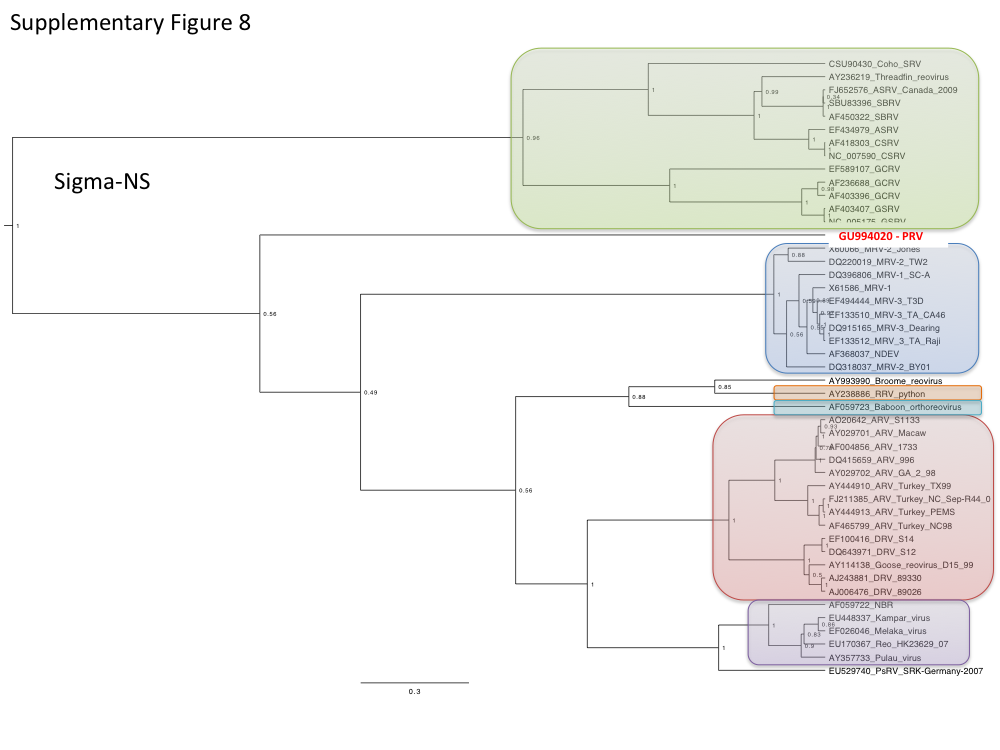

Supplement: Figure S8 — Phylogenetic analysis of the Sigma-NS ORF of the Aquareovirus and Orthoreovirus. For methods and notations, see Figure S1 legend. (3.00 MB TIF) [file pone.0011487.s008.tif]

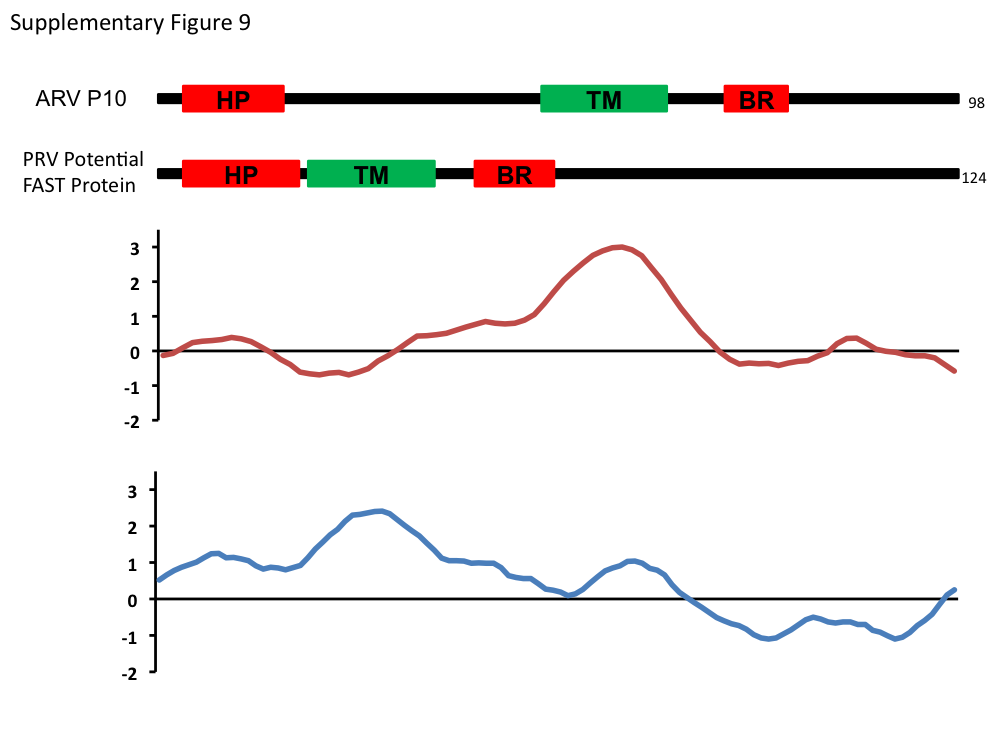

Supplement: Figure S9 — Putative ORF of S1 has characteristics similar to FAST proteins. Hydrophobicity plots of ARV (red) and PRV (blue) obtained using the Kyle-Doolittle algorithm implemented in the program TopPred (available at http://mobyle.pasteur.fr/cgi-bin/portal.py?form=toppred). Sequence analysis show that PRV contains the primary components of a FAST protein: hydrophobic region (HP), transmembrane domain (TM) and basic region (BR). (3.00 MB TIF) [file pone.0011487.s009.tif]
